# Once for All: A Novel Robust System for Co-expression of Multiple Chimeric Fluorescent Fusion Proteins in Plants

**DOI:** 10.3389/fpls.2017.01071

**Published:** 2017-06-20

**Authors:** Guitao Zhong, Qinlong Zhu, Yingxin Li, Yaoguang Liu, Hao Wang

**Affiliations:** ^1^College of Life Sciences, South China Agricultural UniversityGuangzhou, China; ^2^State Key Laboratory for Conservation and Utilization of Subtropical Agro-bioresources, South China Agricultural UniversityGuangzhou, China

**Keywords:** protein co-expression, chimeric fluorescent fusion protein, protein subcellular localization and dynamics, isothermal recombination reaction, transient expression, genetic stable transformation

## Abstract

Chimeric fluorescent fusion proteins have been employed as a powerful tool to reveal the subcellular localizations and dynamics of proteins in living cells. Co-expression of a fluorescent fusion protein with well-known organelle markers in the same cell is especially useful in revealing its spatial and temporal functions of the protein in question. However, the conventional methods for co-expressing multiple fluorescent tagged proteins in plants have the drawbacks of low expression efficiency, variations in the expression level and time-consuming genetic crossing. Here, we have developed a novel robust system that allows for high-efficient co-expression of multiple chimeric fluorescent fusion proteins in plants in a time-saving fashion. This system takes advantage of employing a single expression vector which consists of multiple semi-independent expressing cassettes for the protein co-expression thereby overcoming the limitations of using multiple independent expressing plasmids. In addition, it is a highly manipulable DNA assembly system, in which modification and recombination of DNA molecules are easily achieved through an optimized one-step assembly reaction. By employing this effective system, we demonstrated that co-expression of two chimeric fluorescent fusion reporter proteins of vacuolar sorting receptor and secretory carrier membrane protein gave rise to their perspective subcellular localizations in plants via both transient expression and stable transformation. Thus, we believed that this technical advance represents a promising approach for multi-color-protein co-expression in plant cells.

## Introduction

Green fluorescent protein (GFP) and its variants have been widely used for studying protein localization and dynamics of events such as endomembrane trafficking and cytoskeletal reorganization in living cells. Knowledge on the subcellular localization of proteins provides significant clues for an understanding of their physiological functions and underlying molecular properties (Tsien, 1998; Tsien and Miyawaki, 1998; Zhang et al., 2002; Giepmans et al., 2006; Wang, 2016). In particular, it is useful to co-express a chimeric fluorescent fusion protein with intracellular reporter proteins and organelles in the same cell to better understand its spatiotemporal functions (Lippincott-Schwartz et al., 2001; Zhang et al., 2002, 2004; Tse et al., 2004; French et al., 2008; Lam et al., 2008; Wang et al., 2013, 2016).

**FIGURE 1:**
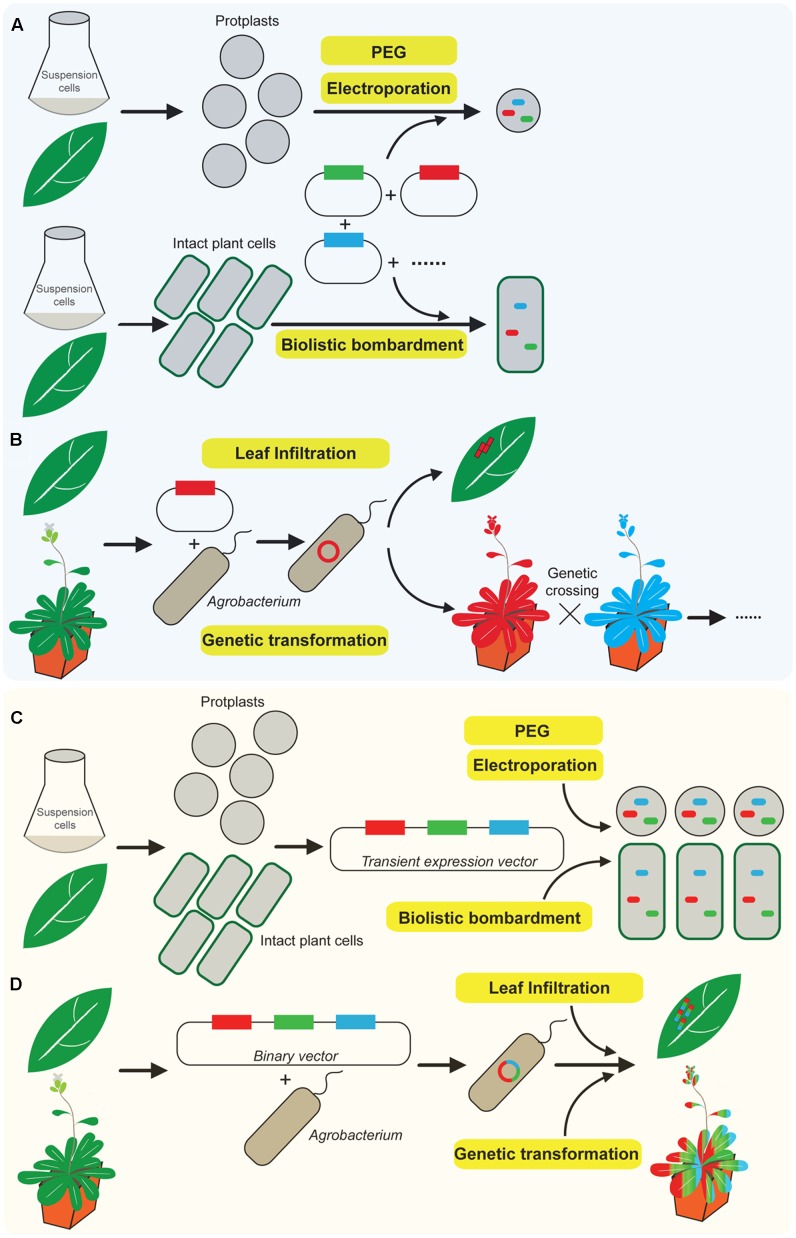
A novel robust system for co-expression of multiple chimeric fluorescent fusion proteins in plants. **(A)** Conventional approaches of co-expression of multiple chimeric fluorescent fusion proteins in plants via transient expression including biolistic bombardment, electroporation, and polyethylene glycol (PEG)-mediated transformation require mixture of several individual plasmids, which dramatically reduce the efficiency of protein co-expression. **(B)** Classical method of *Agrobacterium* mediated either leaf infiltration or stable genetic transformation, the binary vector is usually designed to express a single fluorescent fusion protein at one time. Multiple protein co-expression requires further genetic crossing of individual stable single expression plant. **(C)** Overview of the newly developed novel multiple protein co-expression system. Transient co-expression of multiple chimeric fluorescent fusion proteins via a single transient expression vector in intact plant cells or protoplasts derived from suspension-cultured cells or plant leaves. This was performed either by biolistic bombardment, electroporation or PEG-mediated transformation. In addition, *Agrobacterium* mediated transient expression via tobacco leaf infiltration or stable genetic transformation to express multiple chimeric fluorescent fusion proteins can be achieved via a single binary vector **(D)**.

In plants, the expression of a chimeric fluorescent fusion protein can be achieved via transient expression or stable transformation. Transient expression of fluorescent reporter proteins is an established tool for quickly illustrating organelle dynamics and protein localization (Miao and Jiang, 2007; Martin et al., 2009; Wang and Jiang, 2011; Candat et al., 2013). It has been performed on numerous plant cell types and tissues using various methods of DNA delivery, including electroporation-or polyethylene glycol (PEG)-mediated transient expression of protoplasts derived from suspension-cultured cells or plant leaves, biolistic bombardment and *Agrobacterium* infiltration of tobacco leaves as indicated in **Figures 1A,B** (Miao and Jiang, 2007; Yoo et al., 2007; Wang and Jiang, 2011). However, it requires a mixture of several individual plasmids in order to co-express multiple proteins in a single plant cell as shown in **Figure 1A** (Tse et al., 2004; Nishimura et al., 2006; Lee et al., 2012; Niu and Sheen, 2012; Endler et al., 2015; Zhang C. et al., 2016). Therefore, the efficiency of protein co-expression is relatively low since the successful ratio of co-transforming multiple chimeric fluorescent fusion plasmids in the same cell is lower than one. The transformation efficiency dramatically decreases when the number of co-expressed plasmids is increased. Meanwhile, the expression levels of multiple fusion proteins are difficult to control in the same cell likely due to the random amounts of each plasmid entering into the cell (Zhang et al., 2004; Sainsbury and Lomonossoff, 2014; Canto, 2016).

*Agrobacterium* infiltration of tobacco leaves only mediates the expression of one plasmid at a time. It is therefore technically very challenging to insert multiple expression vectors into one *Agrobacterium* for protein co-expression (**Figure 1B**) (Sparkes et al., 2006; Manavella and Chan, 2009; Canto, 2016). Nevertheless, *Agrobacterium*-mediated stable genetic transformation is an alternative and commonly used approach to express chimeric fluorescent fusion proteins in plants (Varrelmann et al., 2000; Nishimura et al., 2006; Liu et al., 2012; Rosado et al., 2012; Xu et al., 2013; Karnik et al., 2015; Li et al., 2017b). However, the binary expression vector which is carried by *Agrobacterium* for plant transformation is usually designed to express a single fluorescent fusion protein as indicated in **Figure 1B** (Nishimura et al., 2006; Zhang et al., 2006; Ishida et al., 2007; Li et al., 2017a). Thus, it requires further conventional genetic crossing of two different homozygote plants which express two individual fluorescent fusion protein for protein co-expression. It is time-consuming to perform the conventional genetic transformation procedures and subsequent screening for progenies co-expressing multiple fluorescently tagged proteins (Rosado et al., 2012; Karnik et al., 2013; Wang et al., 2014; Zhang M. et al., 2016). Therefore, the development of a convenient, highly efficient and time-saving system for co-expressing multiple chimeric fluorescent fusion proteins in plants via either transient expression or stable genetic transformation will be greatly beneficial and useful to plant biologists.

Here, we present a novel robust system for co-expressing multiple chimeric fluorescent fusion proteins in plants. It differs from and overcomes the various limitations of the previous conventional ones mentioned above. Thus, it would surely significantly speed up studies by applying co-expression of multiple fluorescent proteins in plants. Furthermore, we simplify the procedure of constructing the plasmid and the assembly of DNA molecules by the optimized isothermal *in vitro* recombination assay which conveniently achieves the recombination of multiple DNA fragments in a single reaction.

## Materials and Methods

### Plant Materials, Pollen Tube Germination and Chemicals

The maintenance of suspension culture tobacco (*Nicotiana tabacum*) BY-2 cells was described previously (Jiang and Rogers, 1998; Tse et al., 2004). *Arabidopsis thaliana* (Col-0) seeds were surface sterilized by vortexing mixture with 70% (v/v) ethanol containing 0.05% Tween 20 for 10 min. Spin down the seeds using a bench top centrifuge at max speed for 2 s, remove the supernatant and wash the seeds with 100% ethanol once for 30 s. The seeds were pipetted out onto a sterile filter paper in a sterile hood, air dried and spread on ½ MS agar plates. The plates were first incubated at 4°C for 2 days and then transferred in the plant growth chamber. The settings of growth chamber for Arabidopsis germination and growth: Light intensity: 120–150 μm m^-2^ s^-1^; Temperature: 22°C; Light cycle: 16 h light/8 h dark.

### Plasmids Construction by Optimized Isothermal Recombination Assay

Briefly, the segmented promoter, target gene, fluorescent tag and terminator were amplified using primers (Supplementary Table S1) containing 5′-end overlapping sequence with the adjacent fragment by standard PCR. The DNA templates are derived from previous studies (Wang et al., 2011b, 2016). Then, the first-round PCR products above were mixed and subjected to the optimized isothermal assembly reaction at 50°C for 60 min, followed by amplifying the entire expression cassette using the reaction product as template with the outermost primers (e.g., 1-FP35S and 1-RNOS for expression cassette 1). Finally, the splicing multiple expression cassettes with ends overlapping homologous could be directly assembled into *Sma* I-linearized functional vectors (e.g., pUC18 or pCAMBIA1300) by the optimized isothermal recombination reaction at 50°C for 60 min. The isothermal reaction was adapted and optimized from previous studies (Gibson et al., 2009; Zhu et al., 2014). In general, 400 μl 2× master mixture was prepared by adding 160 μl homemade 5× ISO stock buffer (500 mM Tris-HCl, pH 7.5; 50 mM MgCl_2_; 1 mM dNTP; 50 mM DTT; 25% PEG 8000 and 5 mM NAD) with 3 units of T5 exonuclease (Epicenter), 25 units of Phusion DNA polymerase (New England Biolabs, NEB), 2000 units of *Taq* DNA polymerase (NEB) and sterilized double distilled H_2_O. Aliquot 15 μl per tube and store at -20°C. Add 5 μl mixture of equimolar DNA fragments to 15 μl 2× master mixture for recombination and incubate at 50°C for 60 min.

### *Agrobacterium* Mediated Generation of Transgenic Arabidopsis Plants

The general procedure of *Agrobacterium* transformation was performed as previously described (Clough and Bent, 1998; Tse et al., 2004; Zhang et al., 2006; Manavella and Chan, 2009). In brief, electroporation is used to mediate the transformation of the binary vector into *Agrobacterium* (EHA105). *Agrobacterium* competent cells were thaw on ice for 30 min, then mix the competent cells with 2 μl high-quality plasmid DNA (100–200 ng) which is extracted by plasmid extraction kit (Qiagen) and dissolved in double distilled H_2_O rather than TE buffer. Sit the mixture on ice for 5 min. Transfer the mixture to a pre-chilled 0.1 cm electroporation cuvette (Bio-Rad). Insert cuvette into the Gene Pulser (Bio-Rad) and perform the electroporation with following settings: 25 μF, 1.6 kV, 600 ohms. After the electroporation, add 1 ml super optimal broth with catabolite repression (SOC) immediately into the cuvette and transfer the cells into a 2 ml Eppendorf tube and shake at 28°C, 200 rpm for 120 min. Centrifuge at 6000 rpm at room temperature (RT) for 5 min, resuspend and spread the cells on a LB plate with suitable antibiotics and incubate at 28°C for 2–3 days. To generate the transgenic plants, the resulting constructs were introduced into *Agrobacterium tumefaciens* and transformed into wild-type Columbia-0 by floral dip (Clough and Bent, 1998). The chimeric fluorescent fusion protein co-expression transgenic line was generated using the pCAMBIA1300 plasmid. T0 generations were screened with hygromycin. Seeds were surface sterilized and sown on plates with 1/2 Murashige and Skoog (MS) salts plus 0.8% agar. The seeded plates were kept at 4°C for 3 days before being moved to the growth chamber. The plates were incubated at 22°C under a long-day (16 h light/8 h dark) photoperiod. Seven-day old plants were checked for fluorescent signals under the fluorescent microscope prior to transferred into soil.

### Particle Bombardment

BY-2 cells cultured for 3 days were used for particle bombardment. The sequential procedures for gene delivery into BY2 cells via particle bombardment were the same with the steps for pollen tube bombardment as described before (Wang et al., 2010, 2016; Wang and Jiang, 2011). The bombarded cells were kept in dark in plant growth chamber (27°C) for 6–12 h prior to observation for fluorescent signals. The expression efficiency of chimeric fusion proteins in tobacco BY-2 cells is approximate 5–7% that is consistent with previous studies (Wang and Jiang, 2011; Wang et al., 2011b, 2013).

### Drug Treatment

Stock solutions of wortmannin (1 mM in DMSO; Sigma–Aldrich) and brefeldin A (BFA) (1 mM in DMSO; Sigma–Aldrich) were prepared and stored at -20°C. These drugs were diluted in growth medium to appropriate working concentrations before incubation with plant roots.

### Confocal Microscopy and Colocalization Calculations

In general, the confocal fluorescence images were collected using a Zeiss 710 system with the following parameters: 63× water objective, 700 gain, 0 background, 0.168 mm pixel size, and photomultiplier tubes detector. The images from cells were collected with a laser level of 3% to ensure that the fluorescent signal was within the linear range of detection (typically 0.5% or 1% laser was used). Colocalizations between two fluorophores were calculated by using ImageJ program with the Pearson-Spearman correlation (PSC) colocalization plug-in (French et al., 2008). Results were presented either as Pearson correlation coefficients or as Spearman’s rank correlation coefficients, both of which produce *r* values in the range (-1 to 1), where 0 indicates no discernable correlation while +1 and -1 indicate strong positive and negative correlations, respectively.

## Results

### Development of a Novel System for Co-expressing Multiple Chimeric Fluorescent Fusion Proteins

**FIGURE 2:**
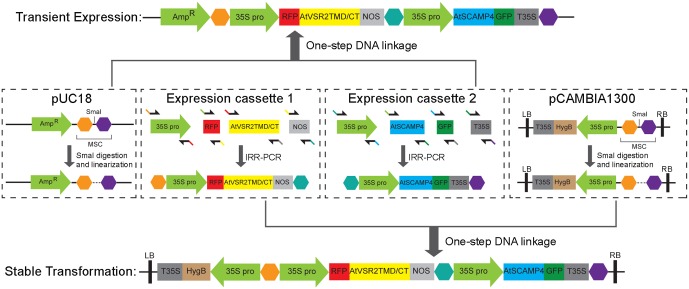
The strategy of constructing a single vector for co-expressing multiple chimeric fluorescent fusion proteins. Schematics of the strategy for constructing a single expression vector for the co-expression of multiple chimeric fluorescent fusion proteins either for transient expression in plant cells or for stable transformation in plants. The expression vector is composed by multiple expression cassettes, each of which contains its own promoter (35S promoter), fluorescent tag (GFP or RFP), target protein (AtVSR2 or AtSCAMP4) and terminator (NOS) respectively, and is responsible for expressing its individual chimeric fluorescent fusion protein. Interlinkage of all the DNA molecules is conveniently achieved by an optimized isothermal *in vitro* recombination method mediated with the overlapping DNA fragments.

We have developed a novel and high-efficient system which differs from the classical approaches (**Figures 1A,B**) for co-expressing multiple chimeric fluorescent fusion proteins in a time-saving fashion (**Figures 1C,D**). It is fully compatible with current approaches of transient expression and stable genetic transformation in plants. In this procedure, we employ a single expression vector which contains multiple protein expression cassettes as shown in **Figures 1C,D**. Each cassette has its own promoter, fluorescent tag, target protein, and terminator, respectively, and is responsible for expressing its individual chimeric fluorescent fusion protein (**Figure 2**). Therefore, it functions semi-independently as a basic “Lego” element and can be manipulated independently according to different expression demands including the requirement for a specific promoter and preference of N- or C-terminal fusion of fluorescent tags with target proteins. Furthermore, all the expression cassettes are linked and integrated into a final single expression vector which is designed for either transient expression or stable plant transformation depending on different experimental requirements.

Additionally, the strategy for assembly of DNA molecules to generate the cassettes for expressing chimeric fluorescent fusion proteins, and the combination of multiple protein expression cassettes with a single destination vector are all conveniently achieved by an optimized isothermal *in vitro* recombination method which is adapted from previous studies (Gibson et al., 2009; Zhu et al., 2014). This takes advantage of homologous recombination of overlapping short DNA sequences to create DNA fusion elements or plasmids with various types of sequence manipulation in one-single reaction. Therefore, ligation of multiple DNA molecules as well as recombination with the destination expression vector are all effectively achieved in one reaction at one time without the need of multiple steps of restriction enzyme digestion and DNA ligation.

Therefore, we have developed at once a robust system for the convenient co-expression of multiple chimeric fluorescent fusion proteins in plants. In addition, it is a highly manipulable “Lego” system that can be easily modified and re-constructed via a one-step DNA junction reaction.

### Co-expression of Vacuolar Sorting Receptor (VSR) and Secretory Carrier Membrane Protein (SCAMP) in Plant Cells

To test this system, we chose two marker proteins: the vacuolar sorting receptor (VSR) and the secretory carrier membrane protein (SCAMP) which participate in the secretory and endocytic pathways, respectively (Tse et al., 2004; Lam et al., 2007a; Wang et al., 2010, 2011a). VSRs are type-I integral membrane family proteins with a single transmembrane domain (TMD) and a short cytosolic tail (CT). VSR mediates the transport of cargo proteins which contain vacuolar sorting determinants (VSDs) to the vacuole via the secretory pathway. The VSR N-terminus is responsible for cargo protein recognition and binding whereas the TMD and CT are sufficient for its correct targeting and localization (Tse et al., 2004; Shen et al., 2014). Subcellular localization studies by confocal immunofluorescent and immunogold EM have shown that VSR proteins are mainly localized to prevacuolar compartments (PVCs) which have been identified as multivesicular bodies (MVBs) in tobacco BY-2 cells (Tse et al., 2004). In contrast, SCAMPs are type-IV membrane proteins localizing to the plasma membrane (PM) and early endosome (EE) or *trans*-Golgi network (TGN) in the endocytic pathway. SCAMP has been used as a reporter protein to follow the endocytic process in plant cells (Lam et al., 2007a). Although the protein secretory pathway and endocytosis are two distinct pathways for protein trafficking and targeting, it has been revealed that TGN serves as a junction for the secretory and endocytic pathways in plant cells (Lam et al., 2007b). Nevertheless, the underlying molecular mechanism regulating the coordination of the two pathways in terms of cell development and morphogenesis remains unsolved. Therefore, co-expression of VSR and SCAMP proteins should provide essential hints toward a better understanding of the spatiotemporal interactions in the secretory and endocytic pathways in plant cells.

**FIGURE 3:**
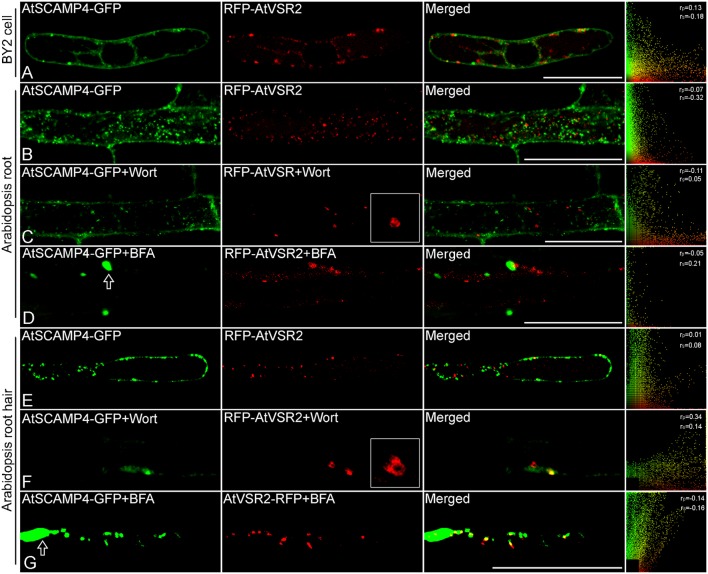
Co-expression of chimeric fluorescent fusion of vacuolar sorting receptor (VSR) and secretory carrier membrane protein (SCAMP) in plant cells. **(A)** Transient co-expression of RFP-AtVSR2 and AtSCAMP4-GFP via biolistic bombardment in tobacco BY2 suspension cells. Scale bar: 50 μm. **(B)** A representative image of a transgenic Arabidopsis root cell expressing RFP-AtVSR2 and AtSCAMP4-GFP via a single binary vector. Scale bar: 50 μm. **(C,D)** Transgenic Arabidopsis plant root expressing RFP-AtVSR2 and AtSCAMP3-GFP after treatment with 16.5 μM wortmannin and 10 μg ml^-1^ brefeldin A (BFA) for 30 min, respectively. Prevacuolar compartment (PVC) enlargement caused by homotypical fusion of PVCs induced by wortmannin is shown in **(C)**, and early endosome aggregation induced by BFA treatment is indicated by arrow in **(D)**. Scale bar: 50 μm. **(E)** A representative image of a transgenic Arabidopsis root hair expressing RFP-AtVSR2 and AtSCAMP4-GFP via a single binary vector. **(F,G)** Transgenic Arabidopsis plant root hairs expressing RFP-AtVSR2 and AtSCAMP4-GFP after treatment with 16.5 μM wortmannin and 10 μg ml^-1^ BFA for 30 min, respectively. PVC enlargement caused by homotypical fusion of PVCs induced by wortmannin is shown in **(F)**, and early endosome aggregation induced by BFA treatment is indicated by arrow in **(G)**. Scale bar in **(E–G)** is 30 μm. The ImageJ program with the Pearson-Spearman correlation (PSC) colocalization plug-in was used to calculate the colocalizations between these two fluorophores. Results are presented either as Pearson correlation coefficients or as Spearman’s rank correlation coefficients, both of which produce *r*-values in the range -1 to +1, where 0 indicates no discernable correlation while +1 and -1 indicate strong positive or negative correlations, respectively.

By applying this newly developed co-expression approach of multiple chimeric fluorescent fusion proteins, RFP-AtVSR2 and AtSCAMP4-GFP were first inserted into two independent expression cassettes as shown in **Figure 2**. All of the DNA fragments in these two expression cassettes were joined via the improved isothermal recombination reaction by short overlapping DNA sequences (**Figure 2** and Supplementary Table S1). Thereafter, the expression cassettes which express RFP-AtVSR2 and AtSCAMP4-GFP, respectively, were linked and integrated into the destination vector pUC18 which is used for transient expression or pCAMBIA for plant stable transformation by applying the same DNA assembly strategy (**Figure 2**). Transient co-expression of RFP-AtVSR2 and AtSCAMP4-GFP in *N. tabacum* BY2 suspension cells was tested by biolistic bombardment. RFP-AtVSR2 gave rise to a punctate pattern as shown in **Figure 1C**. In contrast, AtSCAMP4-GFP localized on the PM with only some cytosolic punctate dots. As shown in **Figure 3A**, the subcellular localization of RFP-AtVSR2 is therefore clearly distinct from that of AtSCAMP4-GFP. In addition, RFP-AtVSR2 and AtSCAMP4-GFP were stably co-expressed in Arabidopsis plants via *Agrobacterium*-mediated genetic transformation. In transgenic root cells and root hairs as shown in **Figures 3B,E**, RFP-AtVSR2 and AtSCAMP4-GFP were successfully co-expressed and their perspective subcellular localizations were consistent with the results obtained from BY2 cells. Moreover, pharmaceutical treatments of the transgenic Arabidopsis roots and root hairs with wortmannin and BFA confirmed that the RFP-AtVSR2-labeled PVC responded to wortmannin by forming ring-like structures (through homotypical fusion of MVBs and AtSCAMP4-GFP-labeled TGN) and responded to BFA (by forming Golgi aggregates) (**Figures 3C,D,F,G**). Therefore, co-expression of fluorescent tagged VSR and SCAMP using this novel protein expression system via either transient expression or stable plant transformation reliably delivers correct subcellular localizations in plant cells (Tse et al., 2004; Lam et al., 2007a; Wang et al., 2010, 2011b).

## Discussion and Conclusion

The development of gene expression vectors and plant transformation technology overcomes the barriers of species and enables introduction of genes into plants from other species or kingdoms. It is broadly used in and staple with plant biotechnology and agricultural industry (Liu et al., 1999; Varrelmann et al., 2000; Qin et al., 2008; Li et al., 2017a). Here, we have developed a highly efficient system which allows for convenient co-expression of multiple chimeric fluorescent fusion proteins in a single plant cell or a whole plant in a time-saving fashion. However, every technology has its advantages and drawbacks. Gene silencing is one group of annoying exceptions when introducing additional copies of a transgene or increasing the gene expression level in plants. Usually, more active and progressive promoter such as cauliflower mosaic virus promoter (CaMV 35S) is employed to enhance the transgene expression instead of using its endogenous native promoter. However, in some instance, the result was not over expression of the gene as expected, but a dramatic reduction of the expression of the introduced gene caused by gene silencing. Under this circumstances, gene silencing appears to be unpredictable with the silencing ratio from 2 to 100% (Dehio and Schell, 1994; Bruening, 1998; Wassenegger and Pelissier, 1998). Gene silencing can be induced by expression of a transgene via both transient expression and genetic transformation. Additionally, gene silencing is more likely to occur in the plants with multiple copies/high-level transcription of the transgene and co-expression of multiple transgenes (Turnage et al., 2002; Golenberg et al., 2009). To minimize the effects of gene silencing in our robust chimeric fluorescent fusion protein co-expression system, avoid continuous usage of the same active promoter in distinct protein expression cassettes when the numbers of co-expressed fusion proteins is more than two. For example, 35S promoter and ubiquitin (UBQ) promoter are suggested to be rotationally used for constitutive co-expressing multiple chimeric fluorescent fusion proteins. Furthermore, the capacitability of the numbers of chimeric fluorescent fusion proteins which can be inserted in the final single expression vector is depending on the backbone plasmid and the size of introduced genes. Different expression vectors such as pCAMBIA1300 and artificial chromosome (TAC) vector with distinct replicon are capable of hosting different size and numbers of insertion transgenes (Liu et al., 1999, 2000).

Taken together, the advantages of the new protein co-expression system are: (i) it employs a single expression vector that is capable of co-expressing multiple fluorescent chimeric fusion proteins thereby overcoming the various limitations of using multiple expression plasmids; (ii) each expression cassettes contained in the single expression vector is semi-independent and can be flexibly manipulated, respectively, according to different expressing demands; (iii) it is a highly efficient and manipulable DNA assembly system, in which recombination of all DNA fragments is simply achieved through an optimized one-step reaction without cutting and joining DNA molecules with restriction enzymes and ligation; and (iv) it is fully compatible with existing fluorescent protein based applications such as fluorescence resonance energy transfer (FRET) and bimolecular fluorescence complementation (BiFC) analysis for illustration of protein–protein interactions. Therefore, this technical advance represents a promising approach for wild aspects of biological discoveries by employing co-expression of multiple chimeric fluorescent fusion proteins in plant cells.

## Author Contributions

GZ and HW conceived with the study; GZ, QZ, and YLi conducted most of the research; GZ, QZ, YLiu, and HW analyzed the data; GZ and HW wrote the manuscript; HW supervised the project.

## Conflict of Interest Statement

The authors declare that the research was conducted in the absence of any commercial or financial relationships that could be construed as a potential conflict of interest.
