# Supplementary material for: Once for All: A Novel Robust System for Co-expression of Multiple Chimeric Fluorescent Fusion Proteins in Plants
Source: Front Plant Sci. 2017 Jun 20;8:1071. doi: 10.3389/fpls.2017.01071 (PMC5476739; doi:10.3389/fpls.2017.01071)
Supplement: Supplementary file 1 [file Table_1.DOC]

**Supplementary Table S1.** Detailed information and primer sequences used in this study.

Name of each primer is given on the left panel. The overlapping sequence of the primers are underlined for DNA recombination.

| ***Expression cassette 1*** | |
| --- | --- |
| 1-FP35S | GAATTCGAGCTCGGTACCCACATGGTGGAGCACGACACA |
| 1-RP35S | ATGCGCACCTTGAAGCGCATTATCACATCAATCCACTTGC |
| 1-FRFP | GCAAGTGGATTGATGTGATAATGGCCCACGCCCGCGTCCT |
| 1-RRFP | ATTATCCATATCACTCCCCAGGCGCCGGTGGAGTGGCGGC |
| 1-FAtVSR2 | GCCGCCACTCCACCGGCGCCTGGGGAGTGATATGGATAAT |
| 1-RAtVSR2 | AAATGTTTGAACGATCGGGATTACAACTCTAGTTGAGAAG |
| 1-FNOS | CTTCTCAACTAGAGTTGTAATCCCGATCGTTCAAACATTT |
| 1-RNOS | GTCGACTCTAGAGGATCCCCTCTAGTAACATAGATGACAC |
| ***Expression cassette 2*** | |
| 2-FP35S | TACGAATTCGAGCTCGGTACACATGGTGGAGCACGACACA |
| 2-RP35S | TTAGGATCGTGTCGTGCCATTATCACATCAATCCACTTGC |
| 2-FAtSCAMP4 | GCAAGTGGATTGATGTGATAATGGCACGACACGATCCTAA |
| 2-RAtSCAMP4 | TCCTCGCCCTTGCTCACCATTAGTGCACGCATCAAGGTCG |
| 2-FGFP | CGACCTTGATGCGTGCACTAATGGTGAGCAAGGGCGAGGA |
| 2-RGFP | TAAAACCAAAATCCAGTGACTTACTTGTACAGCTCGTCCA |
| 2-FT35S | TGGACGAGCTGTACAAGTAAGTCACTGGATTTTGGTTTTA |
| 2-RT35S | CCTTAGGACCGTTATAGTTGGTCCGCAAAAATCACCAGTC |
